# Iodine in Erysipelas and Small-Pox

**Published:** 1883-01

**Authors:** 


IODINE IN ERYSIPELAS AND SNALL-POX.

The *British Medical Journal* has published lately, amongst its therapeutic memoranda, statements commendatory of the action of iodine as a paint in erysipelas. Strangely enough, this old practice was given as if it were something new. After a month or so, the author discovered that he was in error in supposing he was the originator. My earliest recollection of erysipelas was seeing a friend's face painted in this manner. All the medical writers whose works are at hand mention iodine in this connection. Fourneaux Jordan, quoted by Ringer, recommends blisters or iodine. Tanner ("Practice of Medicine") states that "boundary lines may be drawn on the sound skin with tincture of iodine or nitrate of silver." Smith ("On Diseases of Children") thinks it a better remedy for arresting the extension of erysipelas than nitrate of silver. It should be applied to the margin of the sound skin to the distance of two inches. In the 1848 edition of "Dunglison's Practice of Medicine," Dr. Davies, of Hertford, England, is quoted to the effect that the tincture of iodine, diluted with two parts of alcohol, and applied by means of a camel's-hair brush, is an excellent application.

In the issue of the same journal, of September 30, 1882, Dr. Henry Tomkins writes that it is useful in the above-mentioned modes of application, more particularly the one quoted by Dunglison in 1848. In small-pox, too, he advocates its use. Now, long ago, at least thirty years ago, Dr. Crawford, one of the physicians in the Montreal General Hospital, and a lecturer in McGill College, published an article containing the results of his treatment of small-pox cases by iodine paint, and claiming that it prevented the subsequent pitting.—*Canadian Practitioner.*

